# Superabsorbents and Their Application for Heavy Metal Ion Removal in the Presence of EDDS

**DOI:** 10.3390/polym13213688

**Published:** 2021-10-26

**Authors:** Dorota Kołodyńska, Alicja Drozd, Yongming Ju

**Affiliations:** 1 Department of Inorganic Chemistry, Institute of Chemical Sciences, Faculty of Chemistry, Maria Curie Skłodowska University, Maria Curie Skłodowska Sq. 2, 20-031 Lublin, Poland; 2 Analytical Department, Łukasiewicz Research Network—New Chemical Syntheses Institute, Al. Tysiąclecia Państwa Polskiego 13a, 24-110 Puławy, Poland; alicja.drozd@ins.lukasiewicz.gov.pl; 3 Nanjing Institute of Environmental Sciences, Ministry of Ecology and Environment (MEE), Nanjing 510655, China; juyongming@scies.org; 4 Innovative Laboratory for Environmental Functional Materials and Environmental Applications of Microwave Irradiation, South China Subcenter of State Environmental Dioxin Monitoring Center, South China Institute of Environmental Sciences, Ministry of Ecology and Environment (MEE), Guangzhou 510655, China

**Keywords:** superabsorbents, new complexing agents, EDDS, heavy metal ions

## Abstract

Three acrylic-based superabsorbents—TerraHydrogel^®^Aqua (THA), Zeba^®^Hydrogel (ZH) and Agro^®^Hydrogel (AH) were used to investigate the influence of chemical conditions on kinetic and adsorption behavior towards metal ions in the presence of a chelating agent of a new generation called ethylenediamine-*N*,*N*′-disuccinic acid (EDDS). The effects of relevant parameters—mainly including those of sorbent dose, pH of the solution and initial concentration of Cu(II), Zn(II), Mn(II) and Fe(III) complexes with EDDS as well as phase contact time and temperature—on the adsorption efficiency were studied in detail by the static method. The experimental data were also characterized by kinetic and adsorption parameters obtained based on the Langmuir and Freundlich models of sorption as well as the Lagergren, Ho and McKay and Weber–Morris models.

## 1. Introduction

Superabsorbents, commonly known as hydrogels, have been used extensively in numerous branches of industry, among others, in agriculture. Due to a hydrophilic, three-dimensional network, these innovative materials are able to absorb and retain large volumes of water up to thousands of times greater than their own weight. The unique physicochemical properties of superabsorbents, such as large adsorption capacity, high rate of reversible fluid absorbing power, non-toxicity, mechanical strength and chemical and mechanical resistance, have aroused a great, worldwide interest over the past fifty years [1,2,3]. Notably, many investigations have emphasized that hydrogels can help reduce irrigation water consumption, improve fertilizer retention in soil by decreasing application frequency and increase plant growth rates [4,5,6,7]. The intensively reported literature has clearly revealed the slow-release and water-retention properties of superabsorbents. To the best of our knowledge, the three classes of widely used superabsorbent polymers include mainly natural, semi-synthetic and synthetic ones as well as their modifications [8,9,10,11,12,13,14].

In the present paper, commercial hydrogels were used for the adsorption of Cu(II), Zn(II), Mn(II) and Fe(III) complexes with EDDS. Ethylenediamine-*N*,*N*′-disuccinic acid (H_4_edds, EDDS), commercially available as Enviomet^TM^ produced by Innospec Inc (Littleton, CO, USA), is a structural isomer of EDTA. It forms four isomers: S,S; R,R; R,S and S,R. The S,S-isomer of EDDS, based on the naturally occurring amino acid L-aspartic acid, is readily biodegradable. However, the others are partly or completely non-biodegradable. It can be solubilized in water at any ratio. Formation of EDDS complexes with metal ions can be described as follows:

M^2+^ + H*_n_*edds*^n^*^−4^ ⇄ [M(H*_n_*edds)]*^n^*^−2^ (1)

where edds is the ethylenediamine-*N*,*N*′-disuccinic ligand, M is the metal ion and *n* = 0–3.

From the viewpoint of mechanism, metal ions in the formed complexes shown in Equation (1) were assumed to bind two nitrogen atoms and one oxygen atom of each of the four carboxylate groups. The main species, for example, include the following complexes: [Cu(H_2_edds)], [Cu(Hedds)]^−^, [Cu(edds)]^2−^, [Cu(OH)(edds)]^3−^, [Cu(H_2_edds)_2_]^2^, [Cu(H_2_edds)(Hedds)]^3−^, [Cu(Hedds)^2^]^4−^, [Cu(Hedds)edds]^5−^ and [Cu_2_edds], whereas the following complexes: [Zn(H_2_edds)], [Zn(Hedds)]^−^, [Zn(edds)]^2−^ and [Zn(OH)(edds)]^3−^ are reported for Zn(II) [15]. EDDS, as a biodegradable complexing agent of a new generation compared to EDTA, can be used for production of micronutrient fertilizers as an alternative chelating ligand. This indicates that the effective adsorption of metal complexes with a biodegradable complexing agent, EDDS, could function in new slow-release fertilizers of controlled activity. Therefore, the objective of this paper is to investigate the impact of chemical conditions on the kinetic and adsorption behavior of THA, ZH and AH hydrogels towards Cu(II), Zn(II), Mn(II) and Fe(III) ions in the presence of EDDS. Moreover, the main parameters, including mainly the effects of the sorbent dose, pH of the solution and initial concentration as well as phase contact time and temperature, on the adsorption efficiency are to be investigated in detail. Furthermore, kinetic parameters with the kinetic models, i.e., the pseudo first order (PFO), the pseudo second order (PSO) and the intraparticle diffusion (IPD) equations, will also be simulated. Additionally, based on the linear dependence of the Langmuir and Freundlich isotherms, the maximal adsorption capacities and the constants of the studied hydrogels in relation to the complexes of Cu(II), Zn(II), Mn(II) and Fe(III) with EDDS will be further evaluated. The desorption studies, as well as THA, ZH and AH physicochemical characteristics, are also presented.

## 2. Materials and Methods

### 2.1. Materials

The commercial superabsorbents TerraHydrogel^®^Aqua (THA) (Terra, Zielona Góra, Poland), Zeba^®^Hydrogel (ZH) (Agrecol, Mesznary, Poland) and Agro^®^Hydrogel (AH) (EverChem, Łowicz, Poland) were chosen on account of structural differences and used in the study. Their physicochemical properties are presented in Table 1.

The above-mentioned materials have a cross-linked polyacrylic backbone; only ZH has a modified starch matrix. They are characterized by a high absorption capacity of distilled water—up to 380 g H_2_O per 1 g of hydrogel. They have different grain sizes, up to 1.000 mm in the case of AH hydrogel. The optimal operating pH range for the selected hydrogels indicated by the manufacturers is 5–9.

Aqueous solutions of Cu(II), Zn(II), Mn(II) and Fe(III) complexes with EDDS were prepared by dissolving equimolar amounts of the metal salts Cu(NO_3_)_2_∙3H_2_O, Zn(NO_3_)_2_∙6H_2_O, Mn(NO_3_)_2_∙H_2_O and Fe(NO_3_)_3_∙9H_2_O in the EDDS solution. The initial concentration was 1 × 10^−3^ M. The other chemicals, e.g., NaOH and HCl, were of analytical reagent grade, being purchased from (Avantor Performance Materials, Gliwice, Poland). The study also employed deionized water.

### 2.2. Adsorption Experiments

The adsorption of solutions of Cu(II), Zn(II), Mn(II) and Fe(III) complexes with EDDS (in the M(II)/(III):EDDS = 1:1 system) into hydrogels was investigated using a batch system at room temperature with concentrations ranging from 1 × 10^−3^ M to 7 × 10^−3^ M. First, 0.1 g of THA, ZH or AH and 50 cm^3^ of complex solution were placed in a 100 cm^3^ conical flask and shaken mechanically using the laboratory shaker (at 180 rpm with contact time in the range of 1–240 min). pH values were measured using the C-505 pH meter. The procedure was repeated three times. To study the effect of pH values on adsorption, the experiments were conducted at pH 2.0–12.0 with a contact time of 120 min and at 293 K.

The sorption efficiency (S%) and amount of Cu(II), Zn(II), Mn(III) and Fe(III) complexes with EDDS adsorbed on the selected hydrogels were calculated using Equations (2) and (3), respectively:

(2) $$\%S=\frac{\left(C_{0}-C_{t}\right)}{C_{0}}\times 100\%$$

(3) $$q_{t}=\left(C_{0}-C_{t}\right)\times \frac{V}{m}$$

where *C*_0_ is the initial concentration of the solutions of M(II/III) complexes with EDDS (mg/dm^3^), *C_t_* is the concentration of the M(II/III) complexes with EDDS in the aqueous phase at time *t* (mg/dm^3^), *V* is the volume of the solution (dm^3^) and *m_d_* is the weight of the dried hydrogels (g).

The amounts of the metal complexes adsorbed at equilibrium were calculated analogously, using *C_e_* instead of *C_t_*_._

In order to study the adsorption isotherm of EDDS complexes on the polyacrylic superabsorbents, the two adsorption isotherm models, namely those of Langmuir and Freundlich, were applied to study the equilibrium adsorption data, and they are expressed as Equations (4) and (5), respectively [16,17,18,19,20]:

(4) $$\frac{1}{q_{e}}=\frac{1}{K_{L}q_{0}C_{e}}+\frac{1}{q_{0}}$$

(5) $$logq_{e}=\frac{1}{n}logC_{e}+logK_{F}$$

where *q*_0_ is the Langmuir monolayer adsorption capacity (mg/g), *K_L_* is the Langmuir constant related to the free energy of adsorption (dm^3^/mg), *q_e_* is the amount of M(II) or M(III) complex with EDDS sorbed at equilibrium (mg/g), *C_e_* is the equilibrium concentration (mg/dm^3^), *K_F_* is the Freundlich adsorption capacity (mg/g) and 1/*n* is the Freundlich constant related to surface heterogeneity.

The kinetic expressions used for the analysis of sorption data are the pseudo first order (PFO) and second order kinetic (PSO) models [21,22].

The pseudo first order kinetic equation can be written as follows (Equation (6)):

(6) $$\log\left(q_{e}-q_{t}\right)=logq_{e-}\frac{k_{1}t}{2.303}$$

where *q_e_* and *q_t_* denote the amounts of adsorption at equilibrium and time *t* (mg/g), respectively and *k*_1_ is the rate constant of the pseudo first order adsorption (1/min). Based on the plot of *log* (*q*_*e*,__1_−*q_t_*) vs. *t*, the kinetic parameters were calculated.

The pseudo second order model is expressed as Equation (7):

(7) $$\frac{t}{q_{t}}=\frac{t}{q_{e}}+\frac{1}{k_{2}q_{e}^{2}}$$

where *q_e_* and *q_t_* denote the amounts of adsorption at equilibrium and time *t* (mg/g), respectively and *k*_2_ is the rate constant of the pseudo second order adsorption (g/mg min). The kinetic parameters were calculated based on the plots of *t/q_t_* vs. *t*.

The other kinetic equation used for the analysis of the experimental data is the Weber–Morris equation, i.e., the intraparticle diffusion model (IPD), given below [23]:

(8) $$q_{t}=k_{i}t^{1/2}+C$$

where *k_i_* is the intraparticle diffusion rate constant (mg/g min^0.5^) and *C* is the intercept, which reflects the boundary layer effect. The kinetic parameters were calculated based on the plots of *q_t_* vs. *t*^0.5^.

### 2.3. Instruments

The samples were shaken using a laboratory shaker type 357 of constant vibration speed (180 rpm) (Elpin Plus, Lubawa, Poland). The pH was measured using a pH meter, CPI-505, with a glass electrode (Elmetron, Zabrze, Poland). The concentrations of Cu(II), Zn(II), Mn(II) and Fe(III) complexes with EDDS in the filtrate were determined using the spectrometer ICP-OES of 720 ES type (Varian Inc., Palo Alto, CA, USA). The sample introduction system was equipped with a glass Conikal^®^ nebulizer, cyclonic double pass spray chamber and three-channel peristaltic pump. The optimal operating parameters used for determination of the studied elements by the ICP-OES technique are as follows: power, 1200 W; plasma argon gas flow rate, 15 dm^3^/min; auxiliary argon gas flow rate, 2.25 dm^3^/min; nebulizer argon gas flow rate, 0.2 dm^3^/min; pump rate, 12 rpm; analytical wavelengths: 327.395 nm for Cu, 213.857 nm for Zn, 257.610 nm for Mn and 259.940 nm for Fe. Scanning electron microscopy images were obtained using the Quanta 3D FEG microscope with the EDS/EBSD system (FEI). Fourier transform infrared spectra of hydrogels were recorded by the attenuated total reflectance technique (FTIR-ATR), and measurements were made using a FTIR Carry 630 spectrometer (Agilent Technologies, Santa Clara, CA, USA).

## 3. Results and Discussion

### 3.1. Kinetic Studies

**Figure 1:**
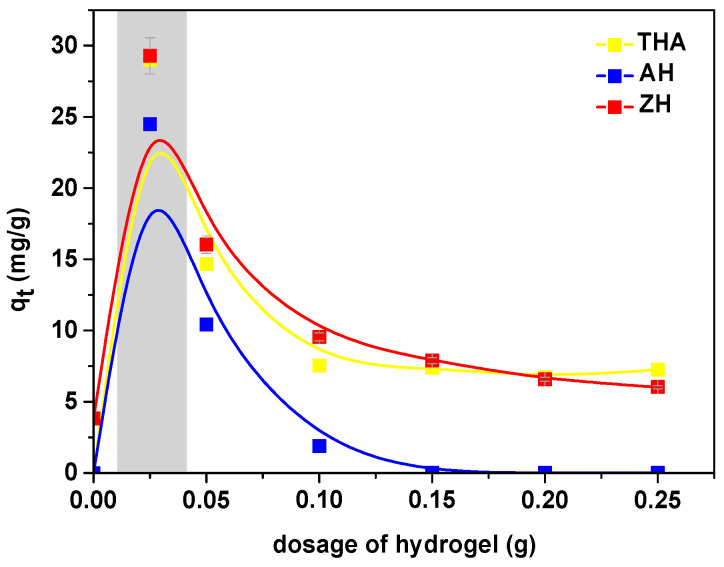
Effect of dosage of hydrogel on the sorption capacity for the Mn(II) complexes with EDDS on AH, ZH and THA (initial concentration 1 × 10^−3^ M).

Adsorption capacities with different dosages of superabsorbents (0.025, 0.05, 0.1, 1.5, 0.2 and 0.25 g) were studied to describe the effect of absorbent dosage and optimize the minimum dosage required for the effective adsorption process of Cu(II), Zn(II), Mn(II) and Fe(III) complexes with EDDS. As an example, the impact of different absorbent dosages is shown in Figure 1 for the Mn(II)–EDDS complexes sorbed on the investigated hydrogels.

**Figure 2:**
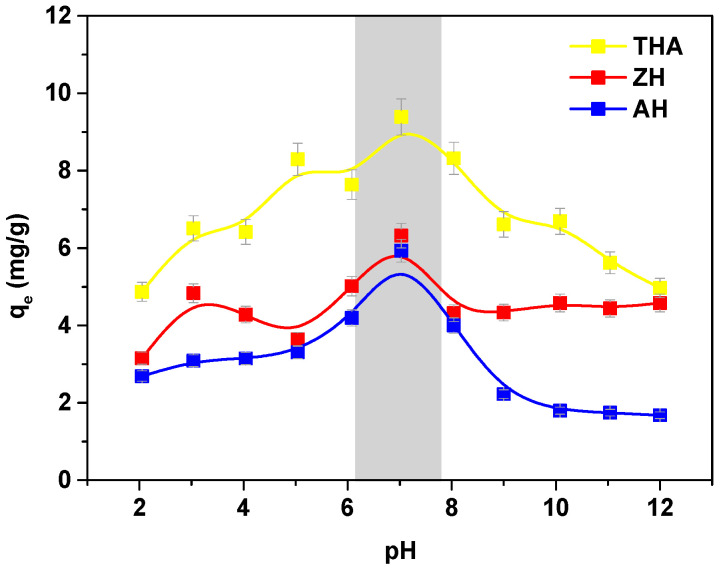
Effect of pH on the sorption capacity for the Cu(II) complexes with EDDS on THA, ZH and AH.

The optimum adsorbent dosage is a key parameter which affects the amount of adsorbed adsorbate. The surface area increases with increasing adsorbent dosage. In this case, the efficiency of the sorption increases; however, the adsorption capacities decreased significantly. The optimum operating range of pH for the selected hydrogels indicated by the manufacturers is 5–9. For comparison of the sorption capacities (*q_e_*) of Cu(II) complexes with EDDS on THA, ZH and AH as a function of pH after 2 h equilibration at various pH values, the obtained results were plotted in Figure 2.

It was shown that during the adsorption process, the pH of the system is altered. Generally, adsorption was more favorable at pH values in the range of 4–10 and dropped sharply at pH values of 2 and 12. pH was adjusted using 1 M HCl and 1 M NaOH.

**Figure 3:**
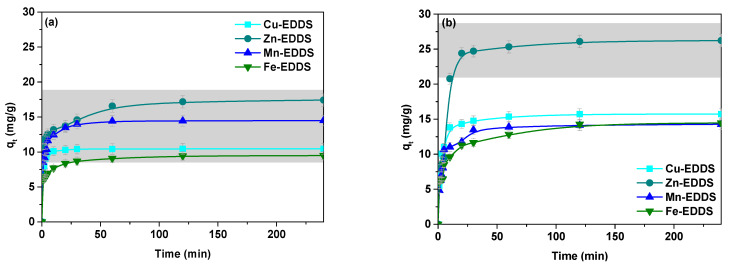

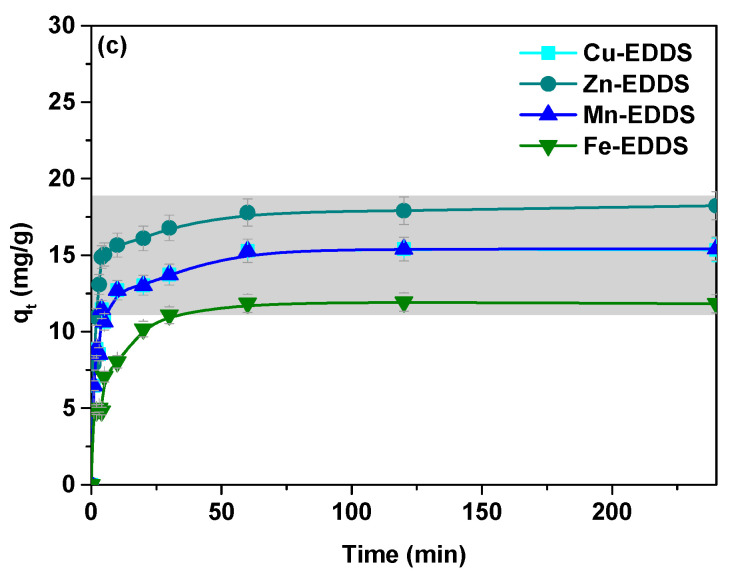
Effect of the phase contact time on the sorption capacity for the Cu(II), Zn(II), Mn(II) and Fe(III) complexes with EDDS on (**a**)THA, (**b**) ZH and (**c**) AH.

The sorption capacity of Cu(II), Zn(II), Mn(II) and Fe(III) in the presence of EDDS was measured at an initial concentration of 1 × 10^−3^ M by varying the contact time in the range of 1–240 min (Figure 3).

Figure 3 shows the obtained results for the sorption of Cu(II), Zn(II), Mn(II) and Fe(III) complexes with EDDS on the THA, ZH and AH hydrogels. It was found that the values of the sorption capacities (*q_t_*) of Cu(II), Zn(II), Mn(II) and Fe(III) in the presence of EDDS on the hydrogels increased with increasing phase contact time and achieved a constant value after about 60 min. The sorption capacities (*q_t_*) for these metal complexes at the initial concentration of 1 × 10^−3^ M after 240 min on the THA hydrogel were: 10.45 mg/g for Cu(II), 17.43 mg/g for Zn(II), 14.49 mg/g for Mn(II) and 9.48 mg/g for Fe(III). The analogous values for ZH were as follows: 15.72 mg/g for Cu(II), 26.23 mg/g for Zn(II), 14.27 mg/g for Mn(II) and 16.46 mg/g for Fe(III). However, for AH they were equal to 15.34 mg/g for Cu(II), 18.23 mg/g for Zn(II), 15.42 mg/g for Mn(II) and 13.83 mg/g for Fe(III). The obtained results indicated that the highest sorption capacity for all investigated hydrogels was found for Zn(II). The other values of sorption capacity are different for each hydrogel. It was found that a more suitable mechanism for the adsorption of Cu(II), Zn(II), Mn(II) and Fe(III) complexes with EDDS on THA, ZH and AH was based on chemical reaction. It involves several steps. The first one is transfer of the solute molecules from the aqueous phase to the area of the solid state, and the second stage is usually a slow process—in the case of solute molecules, diffusion into the interior of the pores. After decomposition in the sorbent phase, metal ions may be attached to the oxygen atoms of carboxylic groups.

The kinetic parameters of the sorption of complexes of Cu(II), Zn(II), Mn(II) and Fe(III) with EDDS on the hydrogels, based on the kinetic pseudo first order (PFO) and pseudo second order (PSO) models recommended by Largergren and Ho, were determined [21,22]. Table 2 summarizes the obtained data. Relatively higher values of the determination coefficients (*R*^2^) were obtained for the kinetic pseudo second order model than the pseudo first order, showing better fit with the experimental data. It was proved that the pseudo second order was optimal for description of the adsorption process. The maximum experimental adsorption capacities (*q_e,exp_*) with respect to the tested complexes, in the case of THA, were as follows: Cu(II)-EDDS, 15.37 mg/g; Zn(II)-EDDS, 16.45 mg/g; Mn(II)-EDDS, 14.47 mg/g; Fe(III)-EDDS, 14.17 mg/g. For ZH, they were: 15.62 mg/g, 16.97 mg/g, 14.45 mg/g and 12.08 mg/g, and for AH: 14.66 mg/g, 22.39 mg/g, 15.99 mg/g and 16.46 mg/g, respectively.

It is well known that intraparticle diffusion plays a significant role in controlling the kinetics of the sorption process. The plots of *q_t_* vs. *t*^0.5^ pass through the origin, and the slope gives the rate constant *k_i_* [22,23]. The values of the rate constants (*k_i_*) and coefficients (*R*^2^) are listed in Table 2. The correlation coefficients (*R*^2^) obtained for this model were smaller and varied from 0.9000 to 0.996. This takes place when the data exhibit multi-linear plots and indicates some degree of boundary layer control. Moreover, it shows that intraparticle diffusion is not the only rate controlling factor but other processes can also control the rate of sorption.

The adsorption capacities of different superabsorbents for uptake of Zn(II) ions, recently reported in the literature, together with that of the ZH hydrogel applied in this study are listed in Table 3.

The hydrogels were studied under different conditions (i.e., initial concentration), and they displayed different adsorption capacities. The maximum adsorption capacity was 274.0 mg/g in the case of the xylene-rich hemicellulose-based hydrogel, and the minimum adsorption capacity of 16.97 mg/g was observed for the ZH hydrogel in this study.

### 3.2. Equilibrium Adsorption Studies

The equilibrium adsorption data are compared according to the linear form of the Langmuir and Freundlich isotherm models. These basic models enable comprehensive explanation of the nature of interactions between the adsorbent and adsorbate. In the Langmuir isotherm model, it is assumed that intermolecular forces decrease rapidly when the distance extends. Another important consideration is the monolayer adsorption onto the surface of the adsorbent containing a specific number of adsorption sites, called active centers. The same affinity for the adsorbate is characteristic of all active centers, with no transmigration of the adsorbate in the surface plane. Additionally, the surface is energetically homogeneous. The essential characteristics of the Langmuir isotherm can also be expressed by the dimensionless constant known as the separation factor *R_L_*_._ For this model, the *R_L_* value indicates that the adsorption nature is either unfavorable (*R_L_* > 1), linear (*R_L_* = 1), favorable (0 < *R_L_* < 1) or reversible (*R_L_* = 0). The *R_L_* values for the studied EDDS complexes on THA, ZH and AH at 293 K were found to be in the range 0.745–0.951 for Cu(II), 0.798–0.878 for Zn(II), 0.812–0.998 for Mn(II) and 0.645–991 for Fe(III). These values indicate favorable sorption of Cu(II), Zn(II), Mn(II) and Fe(III) in the case of EDDS presence on all investigated hydrogels. The relatively low values of the correlation coefficients (*R*^2^) determined from the Langmuir isotherm equation (0.9362–0.9993) indicated that the adsorption process is not homogeneous.

The Freundlich model is widely applied for the heterogeneous surface and for the description of multilayer adsorption. Table 4 shows that the adsorption parameters predicted using the Freundlich isotherm model confirm good agreement with the experimental equilibrium data. In general, as the *K_F_* values increase, the adsorption capacity of the adsorbent increases. In these adsorption studies, the *n* values were found to vary between 0.327 and 1.924. The relatively high values of the correlation coefficients (*R*^2^) determined from the Freundlich isotherm equation (0.9585–0.9979) indicated that the adsorption process is heterogeneous.

The experiments were conducted at three different temperatures (293, 313 and 333 K) in order to study the impact of temperature on the sorption capacity of AH, ZH and THA (Table 4). The complexes’ adsorption decreases slightly with increasing temperature. It can be suggested that this adsorption process is exothermic. For a given constant temperature, the adsorption isotherm describes the dependence of the equilibrium concentration of the studied complexes adsorbed onto THA, ZH and AH on the concentration in the external solution. Considering the above-mentioned relationships, the process can be described from a thermodynamic point of view:

ΔG° = −RT lnK(9)

ΔG° = ΔH° − T ΔS°(10)

where ΔG° is the Gibbs free energy (kJ/mol), ΔH° is the enthalpy (kJ/mol), ΔS° is the entropy (J/mol K), K is the sorption equilibrium constant, R is the universal gas constant (8.314 J/mol K) and T is the absolute temperature (K).

The adsorption equilibrium constant, K, can be obtained from the nonlinear method using Equation (9). If the plot of ΔG° vs. T is linear, the values of ΔH° and ΔS° can be determined from the slope and intercept. If the value of ΔH° is negative, it indicates that the sorption reaction is exothermic and suggests weak binding of the adsorbate to the adsorbent. The calculated negative value of ΔS° indicates greater order of the reaction during the sorption process. The exemplary values of ΔG° calculated for the Mn(II)-EDDS complexes are presented in Table 5.

The enthalpy changes (ΔH°) for the Mn(II) complexes with EDDS sorbed on THA, ZH and AH were −3.08 kJ/mol, −1.77 kJ/mol and −2.93 kJ/mol, respectively. This indicates that the adsorption process is exothermic and the adsorption capacities decrease with increasing temperature. Additionally, according to the heats of sorption, the adsorption processes of Mn(II) complexes on THA, ZH and AH took place by chemical sorption. The negative ΔG° values indicated the spontaneous nature of the adsorption process.

### 3.3. Desorption Studies

After the equilibrium study with the initial concentration of 9 × 10^−3^ M Mn(II)-EDDS, the AH and ZH hydrogels were collected by filtration, dried and transferred to different 100 cm^3^ conical flasks with a 50 cm^3^ desorbing agent and shaken mechanically using the laboratory shaker. One series contained five desorbing agents: distilled water, 0.1 M HCl, 0.1 M HNO_3_, 1 M HCl and 1 M HNO_3_. The samples were agitated at 180 rpm for 24 h.

The concentrations of the eluted Mn(II) ions were measured. The Mn(II) desorption from hydrogels was determined by the following equation:

D% = the amount of desorbed M(II)/amount of adsorbed Mn(II) × 100(11)

The optimal desorption conditions were found using 1 M HCl and 1 M HNO_3_ (Table 6).

### 3.4. THA, ZH and AH Characterization

**Figure 4:**
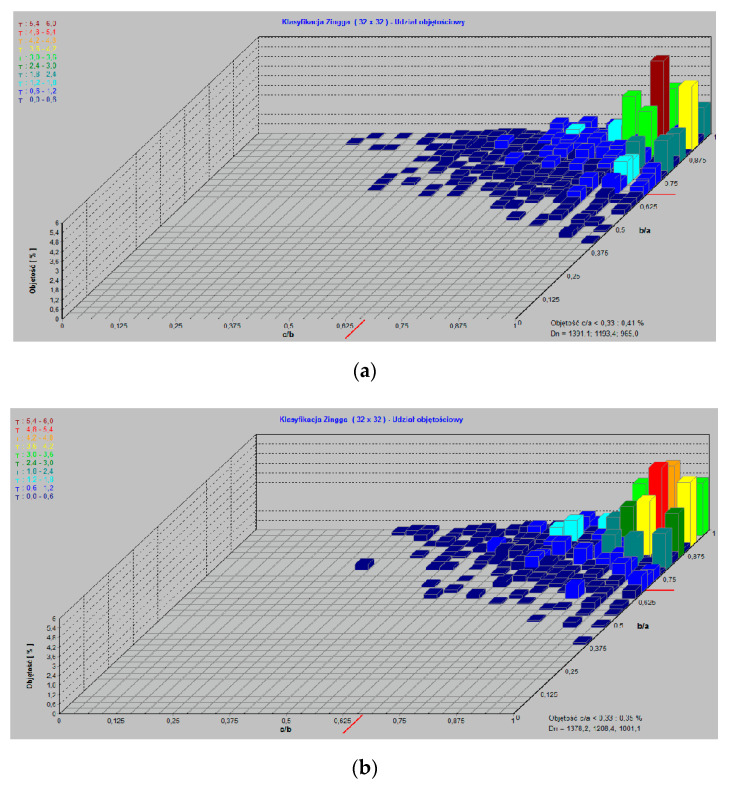

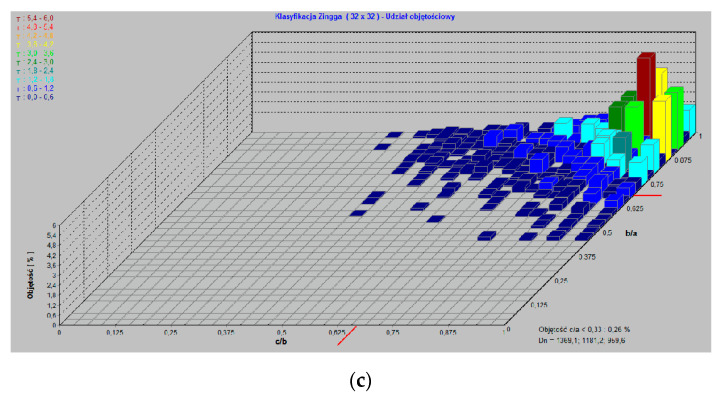
Zinng classification of (**a**) THA, (**b**) ZH and (**c**) THA.

The superabsorbent polymers, THA, ZH and AH, used for the investigations are materials possessing carboxylic acid groups bound to the polymeric matrix. As it was presented in [28], they are characterized by large water absorption and different grain sizes. According to the Zingg clasification [29], more than 62% of hydrogel beads have a spherical shape. It was found that with an increase of bead size, the volume of the disc-shaped fraction decreased in favor of grains in the form of spheres. The obtained results are summarized in Table 7, and the exemplary volume shares of individual beads in the hydrogels are presented in Figure 4.

**Figure 5:**
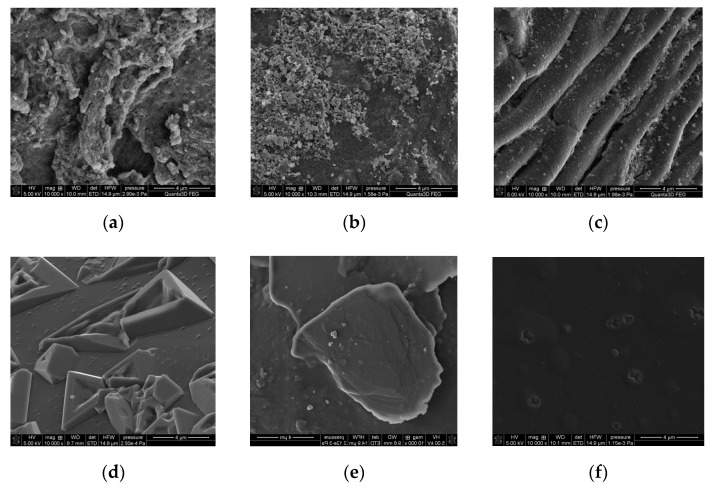
SEM scans of (**a**) THA, (**b**) ZH and (**c**) THA before the sorption process; after the sorption process of the Cu(II) complex with EDDS: (**d**) THA, (**e**) ZH and (**f**) THA.

For example, in the case of AH, the hydrogel bead size is equal to 0.3–1.2 mm, whereas the hydrogel is declared to be 0.17–0.25 mm. The images of THA, ZH and AH obtained from the scanning electron microscope are presented in Figure 5.

As indicated by the analysis of the microscopic scans showing the hydrogels before the sorption process, they differ significantly in their surface morphology. The THA and ZH hydrogels exhibit a clearly loose, rough and granular structure with visible shapeless fragments (Figure 5a,b), in contrast to the AH hydrogel, whose structure is rather compact, folded and regular (Figure 5c,f). In [30,31,32] as well, the dense, smooth surface of the hydrogels was confirmed. As shown by the SEM analysis after the sorption of Cu(II)-EDDS complexes on the above-mentioned hydrogels, the sorbent surface was partially smoothed (Figure 5e,f) [33].

**Figure 6:**
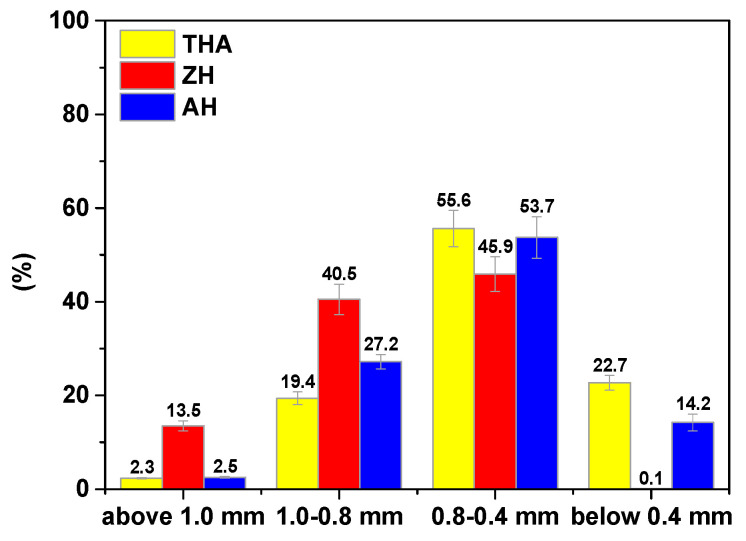
Grain size of THA, ZH and ZH.

They enable accurate determination of the size and shape of individual grains and whole structures. As for the hydrogels, the grain composition diagram indicates that the obtained values of bead size vary compared with those declared by manufacturers (Figure 6). This parameter is important because the larger the grain size, the lower the sorption efficiency.

**Figure 7:**
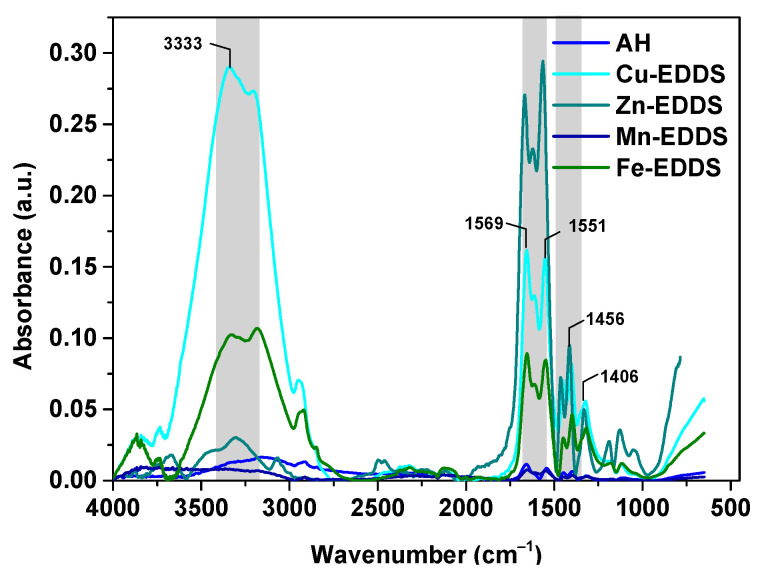
The FTIR-ATR spectra of the AH hydrogel before and after the sorption process of the Cu(II), Zn(II), Mn(II) and Fe(III) complexes with EDDS.

Additionally, the FTIR-ATR spectra of all of the hydrogels were measured before and after the adsorption process of Cu(II), Zn(II), Mn(II) and Fe(III) complexes with EDDS. In Figure 7, the characteristic AH bands, i.e., the stretching vibrations of the -CH_2_ group at 1456 cm^−1^ and the symmetric and asymmetric stretching vibrations of the carboxylate group at 1406 and 1551 cm^−1^, are presented.

**Figure 8:**
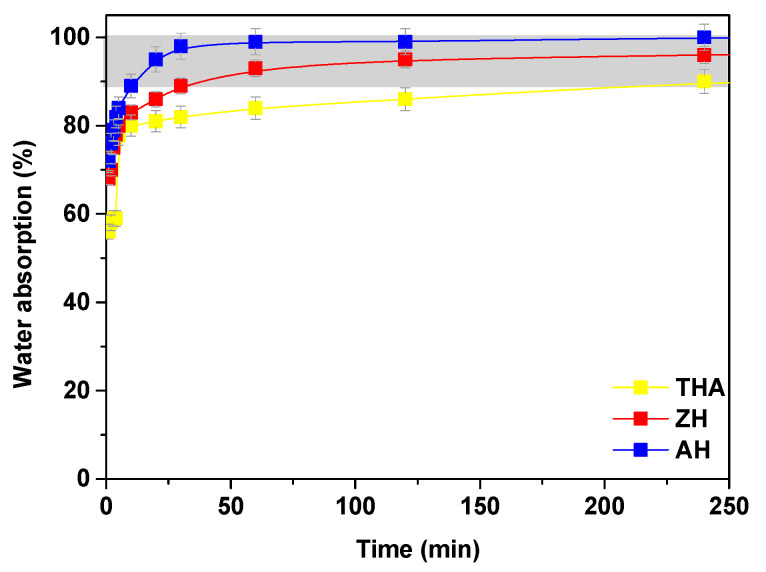
Moisture retention capacity in distilled water for THA, ZH and AH.

**Figure 9:**
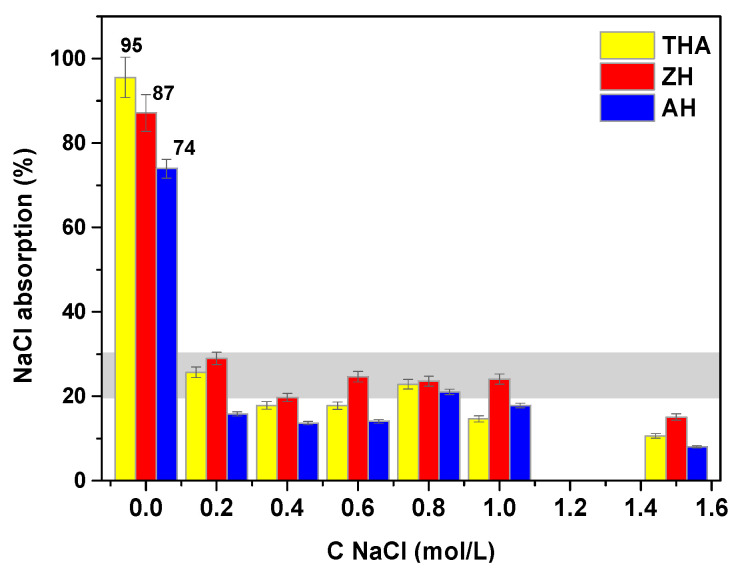
Moisture retention capacity in different concentrations of NaCl solution for THA, ZH and AH.

Another important parameter is swelling. The moisture retention capacities of the investigated hydrogel were determined, and the obtained results are presented in Figure 8 and Figure 9.

Swelling of the hydrogels occurs in several stages. In the first stage, the primary bound water is formed by the hydration of the polar hydrophilic groups of the hydrogel matrix. In the second stage, the secondary bound water is formed by the water interactions with the exposed hydrophobic water. The total bound water includes the primary and secondary bound water. In the third stage, water absorption takes place by the specific interactions between the water osmotic force and the physicochemical cross-links in the polymer network.

All hydrogels exhibit a large water absorbance, approximately 88–100%. The best results are obtained using AH. Moisture retention capacities in the NaCl solution at different concentrations for the AH, ZH and THA hydrogels were also determined. It was proved that NaCl absorption decreases with increasing concentration of NaCl (Figure 9). It was shown that with the increase in its concentration, the retention capacity of the solution in the polymer network decreases and it is much smaller than for distilled water. For the NaCl solutions at concentrations in the range of 0.2–1.6 M, the best results were obtained for the smallest concentrations. At smaller concentrations, the highest absorption capacity was achieved by ZH and the lowest by AH. The swelling capacity analysis reveals that the samples show different absorption due to a varying amount of cross-linking agent. The system becomes more cross-linked due to the presence of cross-links in the hydrogel structure, which increases the density of the network. Shorter segments and smaller voids are formed, and the hydrogel absorbs less solvent. This phenomenon can also be explained by the ion exchange between H^+^ and Na^+^ ions from the NaCl solution, contributing to the reduction of the hydrophilic character of the carboxyl groups as a result of the neutralization reaction [34]. The hydrogel particle surrounded by fluid has a better water absorption capacity, and the diffusion of the fluid is better than in static swelling. As a result, larger masses of swollen gels are obtained.

One of the most important parameters affecting the adsorption capacity is the pH of the adsorption solution. The initial pH of the adsorption medium affects the adsorption mechanisms on the adsorbent surface and influences the nature of the physicochemical interactions of the species in the solution as well as the adsorptive sites of adsorbents.

**Figure 10:**
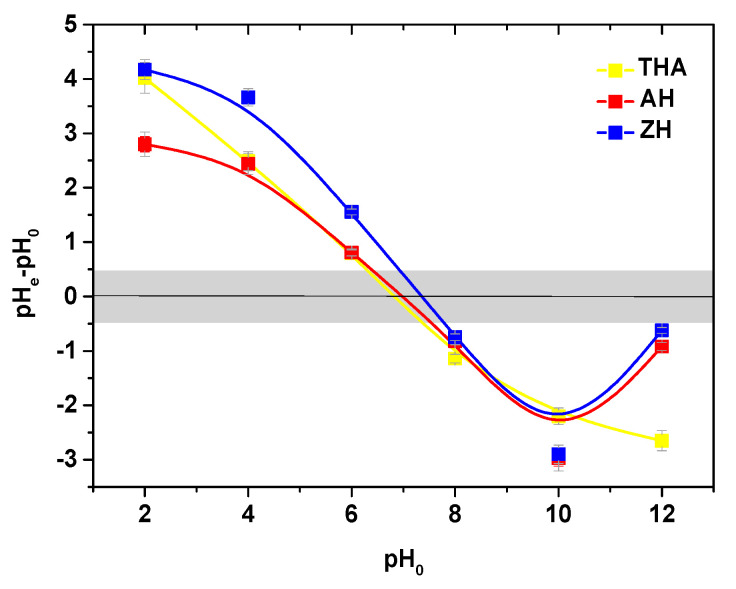
Point of zero charge of THA, AH and ZH.

As follows from Figure 10, the point of zero charge *pH_PZC_* was equal to 6.7, 6.9 and 7.0 for the hydrogels. At pH > *pH_PZC_*, the surface charge will be negative, while it will be positive at pH < *pH_PZC_*.

## 4. Conclusions

The adsorption of the Cu(II), Zn(II), Mn(II) and Fe (III) complexes with EDDS (ethylenediamine-*N*,*N′*-disuccinic acid) onto TerraHydrogel^®^Aqua (THA), Zeba^®^Hydrogel (ZH) and Agro^®^Hydrogel indicates that this complexing agent has a beneficial effect on the efficiency of the adsorption process due to good complexation of metal ions. EDDS is a new generation of complexing agent characterized by a higher biodegradability compared with the conventional agents, such as EDTA. The process efficiency increases with increasing phase contact time, and the adsorption kinetic equilibrium could be reached after 60 min of adsorption. The adsorption process of the Cu(II), Zn(II), Mn(II) and Fe(III) complexes with EDDS onto the selected hydrogels proceeds according to the pseudo second order mechanism reaction, as evidenced by the high values of the determination coefficients. The process is also dependent on pH. The adsorption is more favorable at pH values in the range of 4–10 and dropped sharply at pH values of 2 and 12. The sorption capacities for Cu(II), Zn(II), Mn(II) and Fe(III) complexes with EDDS at the initial concentration of 1 × 10^−3^ M after 240 min on the THA hydrogel were: 10.45 mg/g for Cu(II), 17.43 mg/g for Zn(II), 14.49 mg/g for Mn(II) and 9.48 mg/g for Fe(III). The analogous values for ZH were as follows: 15.72 mg/g for Cu(II), 26.23 mg/g for Zn(II), 14.27 mg/g for Mn(II) and 16.46 mg/g for Fe(III). However, for AH they were equal to 15.34 mg/g for Cu(II), 18.23 mg/g for Zn(II), 15.42 mg/g for Mn(II) and 13.83 mg/g for Fe(III). The adsorption mechanism can be described by the Freundlich equation. The optimal desorption conditions were found using 1 M HCl and 1 M HNO_3._

## Figures and Tables

**Table 1:** The physicochemical properties of THA, ZH and AH hydrogels.

Superabsorbent	THA	ZH	AH
Matrix	acrylic based	modified starch	acrylic based
Cross-linking	polyacrylate	acrylamide–polyacrylate	acrylamide–polyacrylate
Appearance	white	white-yellow	white
Bead size (mm)	0.177–0.255	n.a.	0.300–1.000
Commercial form	Na^+^	K^+^	K^+^
Operating pH range	6–8	5–9	5–9

**Table 2:** The kinetic parameters for the sorption of Cu(II), Zn(II), Mn(II) and Fe(III) complexes with EDDS on AH, ZH and THA.

Hydrogel	M(II)/(III)	PFO			PSO			IPD	
		*q_e_*_1_ mg/g	*k*_1_ 1/min	*R* ^2^	*q_e_*_2_ mg/g	*k*_2_ g/mg min	*R* ^2^	*k_i_* mg/g min	*R* ^2^
THA	Cu(II)	5.43	0.069	0.9626	15.48	0.047	0.9999	4.73	0.9585
	Zn(II)	5.60	0.034	0.7961	16.61	0.025	0.9999	5.21	0.9359
	Mn(II)	4.51	0.073	0.8284	14.54	0.081	0.9999	3.68	0.9799
	Fe(III)	8.61	0.054	0.9248	14.58	0.013	0.9994	3.28	0.9512
ZH	Cu(II)	3.92	0.011	0.9077	15.55	0.019	0.9997	4.22	0.9006
	Zn(II)	5.91	0.033	0.8694	17.15	0.022	0.9999	4.74	0.9473
	Mn(II)	3.26	0.035	0.9287	14.53	0.047	0.9999	4.93	0.9521
	Fe(III)	5.61	0.037	0.8800	12.32	0.019	0.9998	2.78	0.8606
	Cu(II)	4.48	0.074	0.9385	14.74	0.069	0.9999	4.71	0.9962
	Zn(II)	10.51	0.020	0.7056	22.64	0.008	0.9993	4.90	0.9868
AH	Mn(II)	4.48	0.016	0.9523	15.96	0.021	0.9989	4.62	0.9813
	Fe(III)	7.54	0.011	0.7214	16.39	0.009	0.9935	4.44	0.9893

**Table 3:** The comparison of maximum adsorption capacity of different hydrogels for Zn(II) ions.

Hydrogel	*q*_max_ mg/g	References
hydrogel based on chitosan, itaconic and methacrylic acid	105.5	[18]
pH sensitive hydrogel	110	[24]
chelating polymeric hydrogel	83.2	[25]
commercial hydrogel beads	132.5	[26]
xylene-rich hemicellulose-based hydrogel	274.0	[27]
acryloamide polyacrylate (ZH)	16.97 *	this study

* Zn(II)-EDDS complexes.

**Table 4:** The comparison of the maximum adsorption capacities of THA, ZH and AH for the Zn(II) complexes with EDDS.

Hydrogel	Langmuir Model				Freundlich Model		
	*q*_0_	*R_L_*	*K_L_*	*R* ^2^	*K_F_*	1/*n*	*R* ^2^
293 K							
THA	48.69	0.878	0.009	0.9986	1.91	0.48	0.9363
ZH	54.11	0.846	0.012	0.9974	2.23	0.48	0.9191
AH	56.52	0.798	0.016	0.9989	5.20	0.35	0.9499
313 K							
THA	52.12	0.850	0.011	0.9988	2.86	0.43	0.9226
ZH	58.41	0.851	0.011	0.9984	2.41	0.47	0.9245
AH	37.35	0.972	0.018	0.9991	5.59	0.35	0.9434
333 K							
THA	55.78	0.818	0.014	0.9983	5.79	0.34	0.9979
ZH	63.58	0.871	0.010	0.9973	4.21	0.40	0.9906
AH	39.33	0.973	0.018	0.9993	5.71	0.36	0.9397

**Table 5:** Thermodynamic parameters for Mn(II) complexes with EDDS sorbed on THA, ZH and AH.

Hydrogel	T K	ΔG° kJ/mol	ΔH° kJ/mol	ΔS° J/mol K
THA	293 313 333	−14.71	−3.08	−20.3
ZH	293 313 333	−12.95	−1.77	−21.7
AH	293 313 333	−12.14	−2.93	−28.0

**Table 6:** The desorption efficiency of Mn(II) from ZH using different desorbing agents.

Desorbing Agent	Desorption Efficiency %
0.1 M HNO_3_	99.0
0.1 M HCl	97.8
1 M HNO_3_	100.0
1 M HCl	100.0
distilled water	45.0

**Table 7:** The THA, ZH and AH shape characteristics based on the Zinng classification.

Shape	THA [%]	ZH [%]	AH [%]
Sphere	75.38	82.93	75.52
Disc	18.98	12.73	19.21
Rod	5.40	4.15	4.69
Blade	0.24	0.19	0.58

## Data Availability

Not applicable.
